# Editorial: Immunoregulatory cells: balancing immune responses during pathogen infections

**DOI:** 10.3389/fimmu.2025.1664401

**Published:** 2025-07-30

**Authors:** Ana Rosa Pérez, Alexander Batista-Duharte, Gabriel Cabrera

**Affiliations:** ^1^ Instituto de Inmunología Clínica y Experimental de Rosario (IDICER-CONICET-UNR), and CIPREB, Facultad de Ciencias Médicas, Universidad Nacional de Rosario, Santa Fe, Argentina; ^2^ Immunology and Allergy Group (GC01), Maimonides Biomedical Research Institute of Cordoba (IMIBIC), Department of Cell Biology, Physiology and Immunology, University of Cordoba, Reina Sofia University Hospital, Cordoba, Spain; ^3^ Laboratorio de Tecnología Inmunológica, Facultad de Bioquímica y Ciencias Biológicas, Universidad Nacional del Litoral, Santa Fe, Argentina

**Keywords:** FOXP3+ regulatory T cells, myeloid-derived suppressor cells, pathogens, infection, vaccination, sexual dimorphism, Chagas disease, SARS-CoV-2

Over the past century, substantial advances have been made in understanding the role of the immune system in host–pathogen interactions. Numerous humoral and cellular effector mechanisms have been elucidated, supporting strategies designed to enhance immune responses against pathogenic microorganisms. In parallel, the existence and essential function of immunoregulatory cells during infections have gained increasing attention in recent decades (1, 2). Populations such as Foxp3^+^ regulatory T cells (Tregs) (3), myeloid-derived suppressor cells (MDSCs) (4), and other regulatory subsets have progressed from being considered potential actors in specific pathological contexts to being recognized as central players in immune homeostasis across diverse immune scenarios, including infectious diseases (5–7). The growing body of evidence from this complementary area of research has established that immunoregulatory circuits are essential for orchestrating effective immune responses, facilitating robust effector activity while simultaneously limiting collateral damage to self-tissues. Importantly, it has also become evident that certain complex pathogens have evolved mechanisms to dysregulate and even exploit the host’s regulatory immune pathways, thereby interfering and/or suppressing effector responses and promoting their own persistence (8–10). According to this conception, and in order to bridge the gap between studies focused separately on each particular arm of the immune system, this Research Topic has compiled original articles and reviews that provide evidence of the critical interplay between effector and immunoregulatory populations during natural infection, as well as in the context of vaccination or therapeutic approaches.

The severe acute respiratory syndrome coronavirus 2 (SARS-CoV-2) caused one of the most significant global pandemics starting in 2019. The initial absence of an available vaccine and limited understanding of host–pathogen interactions delayed the implementation of effective infection control strategies, resulting in substantial morbidity and mortality (11). Numerous studies have provided evidence that one of the main contributors to the high mortality rates was a dysregulated immune response (12, 13). To provide support to this notion, in this Research Topic Jiménez-Cortegana et al. reported that patients with the worst outcomes exhibited a marked expansion of circulating MDSCs and reduced activation of CD4^+^ and CD8^+^ T cells. These patients also showed persistently low levels of Tregs, suggesting that an immune imbalance could contribute to the severity of the disease. Based on this analysis, which included parameters of both effector and regulatory immune populations, the authors proposed that circulating MDSC levels may serve as a biomarker for predicting the clinical outcome of individuals infected with SARS-CoV-2.

In a different line of research focusing on the virus, Miah et al. reported a strategy that can be used by the virus to leverage the regulatory immune system and attenuate the host’s effector response. The authors identified, through bioinformatic tools and subsequently validated *in vitro*, that the non-structural protein 7 (NSP7) of SARS-CoV-2 contains a Tregitope—an epitope homologous to the previously identified T regulatory cell epitope Tregitope 289. This Tregitope is a naturally occurring T cell epitope derived from the Fc region of human IgG antibody, which specifically activates Tregs, and can cause immunosuppression (14).

This finding aligns with previous work by the research group, describing that although most T cell epitopes found in human pathogens could participate in effector responses, some other specific epitopes could activate Tregs, which can subsequently suppress the host immune response, favoring the persistence of pathogens. The term “immune camouflage” has been proposed to describe the form in which Tregitopes integrated into key antigens may act generating tolerizing responses and thus camouflaging effector or inflammatory epitopes of human pathogens (15). Thus, this study provides new evidence for a recent and additional strategy by which pathogens can leverage human-like epitopes to evade immune responses and potentially induce tolerance toward them.

*Trypanosoma cruzi* (*T. cruzi*) is the etiological agent of Chagas disease, affecting more than 6 million people worldwide, mainly in Latin America (16, 17). Infection by this parasite is well-known to generate an intricate network of host–pathogen interactions, as the parasite has a complex life cycle, exhibits significant genetic variability, and has evolved numerous strategies to infect almost any mammalian cell while evading and manipulating the host immune system (18, 19). For instance, several reports have described how *T. cruzi* delays and distracts the effector immune response stimulating a polyclonal unspecific antibody response (20–22), and also induces the expansion of multiple cell populations with regulatory or suppressive capacity (23). These characteristics may explain, at least in part, why—despite decades of research—no vaccine candidate has yet reached an advanced phase of clinical evaluation (24, 25).

Moreover, despite increasing evidence that immunization impacts not only effector responses but also regulatory cell populations, the role of the regulatory arm of the immune system is seldom considered during immunization and after *T. cruzi* infection (26–28). In this Research Topic, Borgna et al. describe that strategically modulating the involvement of suppressor cells, such as MDSCs, during immunization with a vaccine candidate enhances both the effector immune response and the protective capacity against *T. cruzi*. The study is supported by the use of several preclinical models that differed in mouse strain, parasite strain, dose, and route of infection. This work aligns with some recent studies performed to optimize vaccines against various pathogens, which report that MDSC expansion during immunization may attenuate vaccine efficacy (7). Additionally, the study conducted by Borgna et al. also showed that depleting MDSCs during immunization may subsequently lead to a reduction of these cells during infection, which is paralleled by an increased effector immune response. Accordingly, the study addresses important hurdles in developing a *T. cruzi* vaccine, including genetic variability—since the study was conducted in multiple preclinical models—and the induction of suppressor cells by *T. cruzi*, as vaccinated animals were protected through a mechanism involving lower MDSC increases.

Another important factor that may influence the fine-tuning of the immune system, and is not usually considered in vaccine design in preclinical models, is sexual dimorphism. Balbi et al. addressed this issue in the preclinical evaluation of a vaccine candidate against *T. cruzi*, analyzing differences in both effector and regulatory immune responses in mice that were nasally vaccinated and challenged via the oral route, a particularly relevant approach given that epidemic *T cruzi* food- borne outbreaks are increasing in endemic countries (17). The authors reported that the vaccine elicited sex-specific immunogenicity: while vaccinated female mice developed higher titers of IgG2a, IgG1, and IgA, along with increased production of IFN-γ and IL-17, male mice showed a stronger delayed-type hypersensitivity response and greater proliferation of CD4^+^ IFN-γ^+^ RORγt^+^ T cells. Importantly, despite these differences, vaccinated mice of both sexes were protected against *T. cruzi* infection, as vaccination reduced parasitemia, parasite burden in the heart, intestine, and liver, as well as clinical signs of infection, and significantly decreased acute histological damage in cardiac and muscle tissues. Finally, this study also provided evidence of the beneficial effect of generating a balanced immune response, as vaccinated and infected animals avoided the characteristic expansion of MDSCs, particularly prominent in males, while maintaining Treg frequencies at levels comparable to those of non-infected animals.

The mucosal surfaces are among the sites where immune system balance is critical to support the coexistence of self and commensal microorganisms. The oral microbiota constitutes the second largest microbial community in the human body after the gut. Similar to the gastrointestinal tract, a finely tuned balance between resident microorganisms and the host is essential for maintaining health and preventing disease (29). The review by García-Arévalo et al. compiles current evidence supporting a potential role of the suppressor immune system, particularly focusing on the involvement of MDSCs in various oral pathologies. Although additional research is needed, the findings highlight the relevance of these cells in oral immune regulation.

Bioinformatic analyses have indicated a potential role of MDSCs in oral conditions such as periodontal disease and other inflammatory disorders (30). In addition, since several common oral pathogens such as *Fusobacterium nucleatum*, *Porphyromonas gingivalis*, *Streptococcus mutans*, and various *Candida* species have been shown to induce MDSC expansion *in vitro* and in murine models, the authors emphasize the importance of investigating whether co-occurrence of infection and disease may synergistically drive MDSC expansion, potentially disrupting immune homeostasis in the oral cavity. According to this, the authors propose that a deeper understanding of these mechanisms could have important therapeutic implications for managing oral and systemic diseases (García-Arévalo et al.).

At the systemic level, sepsis is one of the best examples illustrating the importance of the balance between the effector and regulatory arms of the immune system in the context of pathogen challenge. This condition, in which hyperinflammation and immunosuppression play important roles, accounts for nearly 20% of global deaths and is characterized by a dysregulated host response to infection that may lead to multiple organ dysfunction (31). In their comprehensive review, Liu et al. summarize evidence analyzing the dynamic balance of Th17/Treg cells during different phases of sepsis and in different organs. The review also describes the cytokines involved in the differentiation of both populations, highlighting their capacity to transdifferentiate. All this information could be relevant for future therapeutic strategies aimed at fine-tuning the most appropriate balance that enables pathogen control while avoiding both excessive hyperinflammation and excessive immunosuppression. The authors call for further translational studies to define optimal intervention points and validate these findings in clinical settings.

Taken together, the reports included in this Research Topic underscore the importance of understanding the dual nature of the immune system and provide evidence that integrating knowledge derived from the study of both its effector and regulatory arms may guide the implementation of more refined prophylactic and therapeutic strategies. Better control of pathogens will depend on the ability to finely tune the dynamics and specificity of immune responses, promoting maximal specific activation with minimal inflammation and collateral damage, to reach the limits of the system’s protective capacity in each particular setting. In the coming years, significant advances are expected in this critical area, driven by emerging technologies and a deeper understanding of immune regulation.
